# A93 AN ANALYSIS OF POST-ENDOSCOPY EXPERIENCES WITHIN AN EARLY ADULT IBD PATIENT POPULATION AT A NATIONAL IBD CENTRE

**DOI:** 10.1093/jcag/gwad061.093

**Published:** 2024-02-14

**Authors:** M Hu, Z Mohmand, W Wu, N Bollegala

**Affiliations:** University of Toronto Temerty Faculty of Medicine, Toronto, ON, Canada; University of Toronto Temerty Faculty of Medicine, Toronto, ON, Canada; Women's College Hospital, Toronto, ON, Canada; University of Toronto Temerty Faculty of Medicine, Toronto, ON, Canada

## Abstract

**Background:**

Up to 25% of IBD patients are diagnosed before the age of 18, necessitating a process of transition from pediatric to early adult care. Poor endoscopy experiences have been described to jeopardize the overall patient experience with the healthcare system. There is a paucity of literature evaluating the endoscopy experiences of IBD patients during this pivotal point in their disease management.

**Aims:**

The aim of this study is to describe the reported post-endoscopy experiences of early adult IBD patients (18 to 25 years old) compared to those in an adult model of care (26 years old, or above).

**Methods:**

A retrospective analysis of patients with IBD treated at Mount Sinai Hospital from January 1st, 2017 to December 31st, 2021 who underwent an outpatient colonoscopy was performed. Qualitative and quantitative data were longitudinally collected from endoscopy records. The first phase of the study consisted of a comparison of outcomes between early adults (18 to 25 years) and adults (26 years, or above). The second phase consisted of a comparison of outcomes between sedation types (deep versus conscious). T-test was used to compare differences for continuous variables and Fisher exact test was used for categorical variables.

**Results:**

In this initial pilot study of 171 patients, 12.9% of early adults underwent deep sedation compared to 11.4% of adults. Patients who underwent deep sedation were more likely to have Crohn’s disease compared to Ulcerative Colitis (95.2%, 57.7%, pampersand:003C0.01). There were no differences in mean procedure lengths in minutes (18.3, 18.6, p=0.80). Early adults who underwent conscious sedation required additional sedative medications during the procedure in 48.9% of cases compared to 32.3% in the adult cohort (p=0.04), however the mean total of conscious sedation medication used had no differences between the two groups (p=0.59). Nursing post-procedure subjective scoring measuring pain and nausea showed no significant difference (p=0.29). Physician narrative note descriptions of subjective procedural tolerance also showed no significant differences (p=0.78).

**Conclusions:**

Patients with Crohn’s disease are more likely to require deep sedation for their adult colonoscopies compared to patients with Ulcerative Colitis. Gastroenterologists are not adequately prepared to gauge the amount of conscious sedation an early adult patient will require, necessitating top-up amounts peri-procedure more frequently. Current tools in the forms of subjective scoring and narrative documentation utilized by healthcare providers to measure procedural experience and tolerance are poor markers of actual patient experience. Future efforts should be focused on better understanding the endoscopic experience of the early adult IBD patient population and utilizing an innovative and collaborative approach.

**Funding Agencies:**

Division of Gastroenterology, University of Toronto Faculty of Medicine Resident Research Grant

